# *BAGE* Hypomethylation Is an Early Event in Colon Transformation and Is Frequent in Histologically Advanced Adenomas

**DOI:** 10.3390/cancers1010003

**Published:** 2009-11-18

**Authors:** Erica Lana, Marie-Elisabeth Brun, Isabelle Rivals, Janick Selves, Sylvain Kirzin, Andriy P. Lutsyk, Vasily V. Gordiyuk, Frédéric Bibeau, Alla Rynditch, Albertina De Sario

**Affiliations:** 1 INSERM U827, Montpellier, France; E-Mail: erica.lana@inserm.fr (E.L.); 2 Institut de Génétique Humaine, CNRS UPR 1142, Montpellier, France; E-Mail: elisabeth.brun@igh.cnrs.fr; 3 Equipe de Statistique Appliquée, ESPCI ParisTech, Paris, France; E-Mail: isabelle.rivals@espci.fr; 4 CHU Purpan and INSERM U563, Toulouse, France; E-Mail: selves.j@chu-toulouse.fr (J.S.), kirzin.s@chu-toulouse.fr (S.K.); 5 O.O. Bogomoletz National Medical University, Kyiv, Ukraine; E-Mails: alutsyk@gmail.com; 6 Department of Functional Genomics, Institute of Molecular Biology and Genetics NASU, Kyiv, Ukraine; E-Mail: vasilij_gordiyuk@yahoo.com (V.V.G.), rynditch@imbg.org.ua (A.R.); 7 CRLC, Montpellier, France; E-Mail: frederic.bibeau@valdorel.fnclcc.fr

**Keywords:** *BAGE*, DNA hypomethylation, adenomas, colorectal cancer, biomarker, COBRA

## Abstract

We showed earlier that *BAGE* (*B melanoma antigen*) loci are hypermethylated in normal tissues and hypomethylated in 98% of human cancers. More recently, we provided evidence that hypomethylation of *BAGE* loci represents an informative marker for colon cancer detection. In this study, we show that hypomethylation of *BAGE* loci was an early event that occurred in 43% of colorectal adenomas. Interestingly, hypomethylation of *BAGE* loci was frequent (50%) in tubulo-villous and villous adenomas, these adenomas having a high probability of being transformed into colorectal cancers.

## 1. Introduction

Colorectal cancer is the third most common cancer worldwide and is the third leading cause of cancer-related deaths. Currently detection of precancerous lesions and cancers at an early stage seems to be the most effective means of reducing mortality. Several colorectal screening tests are available. Fecal occult blood testing, a noninvasive test used to screen asymptomatic subjects that are 50 years or older, has a limited sensitivity [1]. Colonoscopy and sigmoidoscopy are more sensitive, but cannot be used routinely because of high costs and discomfort for the patient. Therefore, optimized screening methods should be established to monitor the general population.

Colorectal cancer results from a multistep process comprising several histological changes, the so called adenoma-carcinoma progression sequence of events [2]. Both genetic and epigenetic mutations associated with these histological changes have been described. Some of these mutations clearly contribute to the transformation process; for others, a pathogenic role is not yet proven. Genetic and epigenetic mutations that are frequently associated with cancer cells can be used as biological markers for cancer diagnosis. Mutations that are already detected in precancerous lesions are the most valuable tumor markers because they allow patients to be treated before they develop metastases.

*BAGE* loci are a family of full-length and truncated genes located in the juxtacentromeric heterochromatic regions of several human chromosomes [3,4]. *BAGE* genes are silent in normal tissues and expressed in some tumors and in male germ cells, whereas truncated genes are nonfunctional. We showed earlier that *BAGE* loci (i.e. genes and truncated genes) are hypermethylated in normal tissues and hypomethylated in 98% of human cancers [5]. More recently, we have shown that *BAGE* loci are hypomethylated in 83% of colon cancers. Using the methylation of *BAGE* loci as tumor marker, colon cancer could be diagnosed with 94% specificity, 83% sensitivity, and 89% accuracy [6]. In the present study, we asked whether *BAGE* hypomethylation can be used to detect colorectal precancerous lesions.

## 2. Results and Discussion

### 2.1. BAGE loci Are Hypomethylated in Colorectal Adenomas

**Figure 1:**
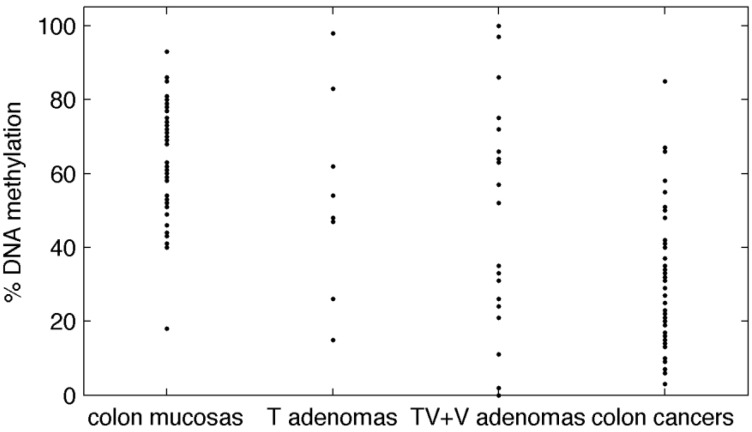
DNA methylation percentages in five tissues. Data on paired colon cancer and healthy mucosas are from our previous study [6]; whereas data on adenomas are from this study (a different cohort of patients). T are tubulous, TV are tubulo- villous and V are villous adenomas.

We showed earlier that *BAGE* loci are hypermethylated in normal tissues and hypomethylated in 83% of colon cancers [6]. To determine whether loss of methylation of *BAGE* loci is an early event in colon transformation, we have analyzed 44 colorectal adenomas (Table 1 and Figure 1) using the COBRA assay that we described earlier [5]. We showed previously that a DNA methylation threshold of 42% made it possible to distinguish colon cancer from normal mucosa with 94% specificity and 83% sensitivity [6]. In the present study, using the same threshold, we found that *BAGE* loci are hypomethylated in 19 out of 44 (43%) adenomas (Table 1 and Figure 1). The percentage of precancerous cells in our samples ranged from 60% to 100% (Table 1). DNA hypomethylation did not correlate with the percentage of precancereous cells observed in adenomas (Spearman test r = −0.01, p = 0.97). Our samples were collected in two different geographical areas: France and Ukraine. In spite of possible ethnic differences in the two patient populations, the prevalence of *BAGE* hypomethylation in colon adenomas was similar: 42% (n = 24) in France and 45% (n = 20) in Ukraine (Fisher’s exact test, two-sided, p = 1). To conclude, our results show that loss of methylation in *BAGE* loci is an early event in colon transformation.

No correlation was found between DNA methylation and age (Spearman’s r = −0.04, p = 0.82) and sex (Wilcoxon test, p = 0.63) of patients, nor with the location of the adenomas (Kruskal-Wallis test, p = 0.76).

**Table 1:** Clinical features of the analyzed samples.

Samples	Sex	Age	Site	Histological type	Grade of dysplasia	% precancerous cells	% DNA methylation^†^
T1	M	62	Right colon	T	LG	60	62
T3	M	75	Sigmoid	TV	LG	100	52
T4*	F	69	Right colon	TV	LG	100	0
T5*	F	69	Right colon	TV	LG	100	24
T6^‡^	M	70	Sigmoid	V	LG	100	63
T7^‡^	M	70	Sigmoid	TV	LG	100	66
T10	M	56	Right colon	TV	LG	60	97
T11	M	63	Left colon	TV	LG	100	11
T12	F	51	Rectum	V	LG	80	0
T13	F	62	Left colon	T	LG	100	83
T14	F	19	Coloproctectomy	T	LG	100	54
T15	F	19	Coloproctectomy	TV	LG	100	2
T16	M	60	Sigmoid	T	LG	100	47
T17	F	56	Coloproctectomy	T	LG	100	98
T18	M	70	Right colon	T	LG	100	48
T19	M	67	Coloproctectomy	V	LG and HG	100	75
T20	M	59	Coloproctectomy	T	LG	100	15
M21	F	56	Colon	V	LG	90	57
M22	F	58	Colon	TV	LG	85	52
M24	M	60	Colon	TV	HG	90	31
M25	M	78	Colon	T	LG	85	48
M26	M	61	Rectum	V	LG	90	33
M27	M	70	Colon	V	HG	90	24
M40	F	21	Colon	T	LG	90	26
K3	M	80	Rectum	–	LG	n.d.	28
K4	F	56	Rectum	V	LG	n.d.	26
K8	M	63	Rectum	–	LG	n.d.	6
K9a#	F	48	Rectum	–	LG	n.d.	50
K9b#	F	48	Rectum	–	LG	n.d.	16
K10	M	61	Rectum	–	HG	n.d.	49
K11	M	63	n.d.	–	LG	n.d.	102
K13	M	39	Rectum	–	HG	n.d.	59
K14	M	84	Rectum	V	HG	n.d.	86
K15	M	53	Rectum	–	LG	n.d.	40
K16	M	46	Rectum	–	LG	n.d.	93
K17	M	67	Rectum	–	LG	n.d.	30
K18	M	72	Rectum	–	LG	n.d.	37
K19	M	58	Rectum	–	LG	n.d.	43
K20	M	28	Rectum	–	HG	n.d.	82
K21	F	56	Sigmoid	V	LG	n.d.	64
K22	F	37	Rectum	V	LG	n.d.	100
K23	M	59	Sigmoid	V	LG	n.d.	21
K24	F	73	Sigmoid	V	LG	n.d.	72
K25	F	77	Rectum	V	LG	n.d.	35

T = tubulous; TV = tubulo-villous; V = villous; LG = low grade; HG = high grade; † Mean of two duplicates; n.d. = not detailed by pathologist; *‡# Two polyps from a same patient; – excluded.

### 2.2. Hypomethylation of BAGE Loci and Histological Features

The prevalence of DNA hypomethylation in *BAGE* loci was higher in tubulo-villous and villous adenomas (50%) than in tubulous adenomas (22.2%) (Table 2-a). Such a difference was not significant (p = 0.15); however, the comparison between these two groups of tissues should be done in a larger number of samples. We analyzed 31 adenomas out of 44: 13 samples were excluded because they had been classified by the pathologist in two groups (tubulous and villous) instead of three (tubulous, tubulo-villous and villous) and we thought this might introduce a bias. No correlation was found between the grade of dysplasia and loss of DNA methylation (Wilcoxon test, p = 0.52). However, most adenomas had a low grade of dysplasia.

**Figure 2:**
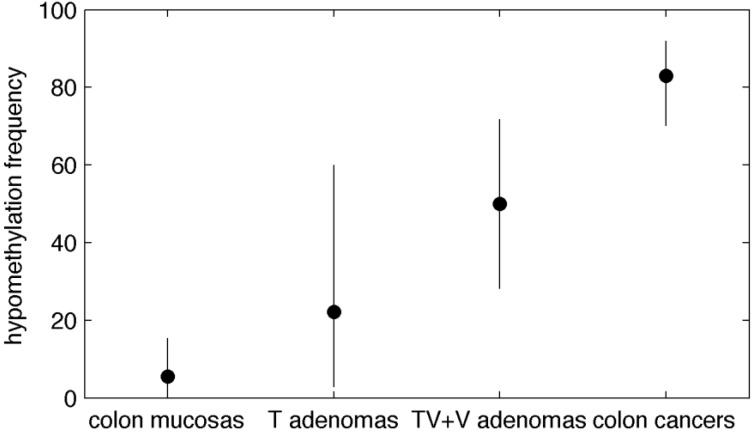
Frequency of hypomethylated DNA (<42% according to [6]) and their 95% confidence intervals. Data in healthy mucosas and colon cancers are from [6]. T are tubulous, TV are tubulo- villous and V are villous adenomas.

Afterwards, we compared the hypomethylation frequency in colon adenomas (data from this work) with the hypomethylation of *BAGE* loci in colon cancers and healthy colon mucosas (data from our earlier study [6]). Paired colon cancers and healthy mucosas were obtained from a different cohort of patients with respect to adenomas. Raw data are represented in Figure 1. The prevalence of hypomethylation was very low in healthy colon mucosas (5.6%), increased in colon adenomas (43.2%), particularly in tubulo-villous and villous adenomas (50%), and rose even higher in colon cancers (83.0%) (Table 2-a; Figure 2). When we compared these tissues two by two, the enrichment in hypomethylated samples was significant in cancers versus adenomas in general and also in tubulo-villous and villous adenomas versus healthy mucosas (Table 2-b). Confidence intervals were wider in adenomas than in colon cancers or mucosas: this variability points up the heterogeneity of DNA methylation in colon adenomas which represent a transition between normal and frankly malignant tissues.

Table 2

**Table 2:** (a) Hypomethylation frequency in various tissues.

Tissues	Frequencies	Min% (2)	Max% (2)
mucosas (1)	(3/54) 5.6%	1.2	15.4
cancers (1)	(44/53) 83.0%	70.2	91.9
adenomas	(19/44) 43.2%	28.3	59.0
T adenomas	(2/9) 22.2%	2.8	60.0
TV + V adenomas	(11/22) 50.0%	28.2	71.8

**Table 2:** (b) Enrichment test of hypomethylated samples.

Tissues	p-Values
adenomas vs mucosas	7 × 10^−5^
cancers vs adenomas	1.4 × 10^−6^
TV+V adenomas vs mucosas	2.7 × 10^−5^
TV+V adenomas vs cancers	4.7 × 10^−3^
T adenomas vs mucosas	0.15
T adenomas vs cancers	6.1 × 10^−4^
TV+V adenomas vs T adenomas	0.15

(1) data from [6]; (2) 95% confidence interval; T = tubulous; TV = tubulo-villous; V = villous.

## 3. Experimental Section

### 3.1.DNA Samples

Forty-four colorectal adenomas were obtained from 40 patients (Table 1). All subjects provided written informed consent. Frozen tissues were used partly for DNA extraction and partly for histopathological inspection. DNA was quantified spectrophotometrically (Thermo Scientific NanoDrop 1000 Spectrophotometer, Wilmington, DE, USA): samples having a ratio A_260/280_ ≥ 1.8 were used for the DNA methylation analysis.

### 3.2.DNA Methylation Analysis

DNA methylation in *BAGE* loci was analyzed according to a previously described combined bisulphite restriction assay (COBRA) protocol [5]. Briefly, genomic DNA was treated with sodium bisulphite and *BAGE* loci were amplified in two consecutive PCR with a set of nested primers and 5 U of Taq DNA Polymerase (MP Biomedicals, Illkirch, France). Primers in the first PCR were BAGE.BS+32f (TttagaggaTTaggagaagggggagT) and BAGE.BS+1540r (AcctAccaAttAAcattAttActAacattA). Primers in the nested PCR were BAGE.BS99f (gatggtggtggTaaTagagatggT) and BAGE.BS+1371r (ccttaAAcaAtAtaAaccctAataA). In the first PCR we used approximately 15 ng of genomic DNA as template, whereas in the nested PCR, we used 2.5 μL of the first PCR mixture. The PCR program was 94 °C for 2 min, followed by five cycles of (94 °C for 1 min, 55 °C for 2 min and 72 °C for 3 min). Then 25 cycles of (94 °C for 0.5 min, 55 °C for 2 min and 72 °C for 1.5 min and a final elongation at 72 °C) for 10 min. The products of three nested PCRs were purified using the QIAquick PCR Purification kit (QIAGEN, Hilden, Germany) and quantified spectrophotometrically. The purified PCR products (700 ng) were then digested with 2.5 U of *Mbo*I enzyme (New England Biolabs) 2 hours at 37 °C and separated by electrophoresis on a 2% agarose gel in SB buffer [8.2 mM NaOH, 5 mM borate]. Images were captured with an Infinity 3000 imaging system (Vilber Lourmat, Marne-la-Vallée, France). This COBRA assay is based upon simultaneous amplification and digestion of several *BAGE* loci that produces a complex pattern of DNA bands. Two bands of 287 bp and 241 bp are diagnostic: their relative intensity is correlated with *BAGE* methylation. The relative intensity of the diagnostic bands was measured in individual samples and in each point of a standard curve that allowed us to calculate the methylation percentages. The standard curve was obtained by combining increasing amounts of sperm DNA (approximately 0% DNA methylation) and *M.Sss*I-treated (New England Biolabs) DNA (100% DNA methylation). All experiments were done in duplicate. Methylation values over 100% have two explanations. First, polymorphism and/or chromosome rearrangements in the samples may change the relative amount of diagnostic bands. Second, the method used to analyse *BAGE* methylation has an accuracy of approximately 10% and this may also account for methylation values over 100%. For a detailed explanation of the method see [5].

### 3.3. Statistical Analysis

To establish whether DNA methylation was correlated with the age of the patients and with the percentage of precancerous cells (continuous variables), we computed Spearman’s correlation coefficient and the associated p-value. To test the correlation with sex and dysplasia (categorical variables), Wilcoxon’s ranksum test was performed. The correlation between DNA methylation and the location of adenomas (right/left colon, sigmoid, rectum and coloproctectomy) was analyzed with a Kruskal-Wallis test. Since the number of samples in each of the five tissues was small, the confidence intervals for the probabilities of Table 2-a and Figure 2 were established using the binomial distribution, and are hence asymmetrical. Probabilities were compared with Fisher’s exact test, either one-sided for the enrichment tests of Table 2-b, or two-sided (see [7] for details). A p-value of less than 0.05 was considered significant.

## 4. Conclusions

Tumor transformation is frequently associated with epigenetic changes: both a global loss of DNA methylation and local increases of DNA methylation in the promoter region of several genes have been reported. Aberrant methylation may contribute to tumor progression by (i) silencing tumor suppressor genes; (ii) activating oncogenes; and (iii) promoting genome instability [8,9]. Detection of aberrant DNA methylation can be used to develop new biological markers that differentiate cancer cells from normal cells [10]. To date, most studies have focused on DNA hypermethylation: several gene promoters are hypermethylated in colorectal cancers and in colorectal adenomas [11,12,13]. Hypermethylation has been found not only in individual genes, but also in large chromosome regions [14]. Few studies have investigated DNA hypomethylation in colon cancer. Bariol *et al*., [15] analyzed global DNA methylation levels in a collection of adenomas, hyperplastic polyps, colorectal cancers, and normal mucosa: normal mucosa was more methylated than any cancerous or precancerous lesion. Overall, these findings show that epigenetic changes occur early during tumor transformation.

We showed earlier that *BAGE* loci were significantly hypomethylated in colon cancers compared to paired healthy mucosas [6]. In the present study, we provide evidence that hypomethylation of *BAGE* loci was an early event occurring in 43% of colorectal adenomas. Interestingly, hypomethylation of *BAGE* loci was frequent (50%) in tubulo-villous and villous adenomas, these adenomas having a high probability of being transformed into colorectal cancers [16]. In conclusion, hypomethylation of *BAGE* loci could be used for the diagnosis of colorectal cancers and histologically advanced adenomas. Aberrant hypermethylation of some genes is also more frequent in tubulo-villous and villous adenomas than in tubulous adenomas. Petko *et al*. [17] observed an increase of hypermethylation in the *CDKN2A* and *MGMT* genes, but not in the *MLH1* gene in advanced adenomas.

Aberrant methylation of gene promoters may be detected in fecal DNA of patients with colorectal carcinomas and adenomas [17,18,19]. In colorectal cancers, the rate of colonocyte exfoliation is much higher than in normal mucosa [20]. Exfoliated cells migrate along the gut following the fecal flow and their DNA can be purified from stools. In spite of the presence of normal contaminating squamous cells, significant changes in DNA methylation at gene promoters have been reported [17,18,19]. The main drawback of using stool DNA to screen the general population is the low sensitivity of single tumor markers. To increase the final sensitivity and specificity, reliable diagnostic assays should combine multiple epigenetic and genetic markers that have been accurately chosen from among the most informative. Likely, a combination of markers based on hyper- and hypomethylation will be more informative because the two events seem to be independent [15]. Among markers based upon loss of methylation, *BAGE* loci are particularly informative. Contrary to LINE sequences that are hypomethylated in 30% of normal mucosa, *BAGE* loss of methylation is very specific and also rather sensitive in DNA that has been extracted from biopsies [6]. Thus, the feasibility of detecting *BAGE* hypomethylation in stools of patients with colorectal cancer and/or precancerous lesions should be evaluated. Another factor to be considered in the development of noninvasive tests to monitor the general population is their cost. To reduce the cost, it will be important to optimize an analytical approach that can be easily implemented in a hospital laboratory and allows the simultaneous analysis of several samples. Pyrosequencing, revealing both point mutations and methylation changes over 100bp-DNA stretches, seems to us an appropriate solution [21]. Kits enabling DNA extraction from stool and bisulfite treatment of genomic DNA can be purchased. Moreover, pyrosequencers host 96 samples per run. This technology, widely used to detect k-ras mutations in cancer, may become a powerful and cost-effective screening method, provided multiple genetic and epigenetic markers are analyzed.
